# Development and biomechanical validation of a whole spine–thorax finite element model for quantitative biomechanical analysis

**DOI:** 10.7717/peerj.20961

**Published:** 2026-03-18

**Authors:** Junhua Li, Yaoshuai Yu, Yuanxun Lin, Hongwen Liu, Weixing Zhong, Lixin Tang, Diangu Chen, Yongliang Ye, Xiaoguang Lin, Tianzhao Tian, Yikai Li

**Affiliations:** 1 The Affiliated Traditional Chinese Medicine Hospital, Guangzhou Medical University, Guangzhou, China; 2 School of Traditional Chinese Medicine, Southern Medical University, Guangzhou, China; 3 The Third Affiliated Hospital of Southern Medical University, Guangzhou, China

**Keywords:** Finite element model, Manual therapy, Spine–thorax complex, Model validation

## Abstract

**Objective:**

To develop a high-fidelity three-dimensional finite element model of the whole spine–thorax complex based on high-resolution computed tomography (CT) images of a healthy adult male, and to perform initial validation under representative loading conditions for quantitative analysis of load transmission, coupled motion, and stress distribution. We hypothesized that the model would reproduce published quasi-static segmental moment–rotation behavior and cadaveric thoracic impact responses within acceptable error ranges.

**Methods:**

High-resolution CT data of one healthy adult Chinese male volunteer (25 years; 175 cm; 70 kg) were used to reconstruct detailed anatomical structures, including vertebrae, intervertebral discs, ribs, costal cartilage, sternum, ligaments, respiratory muscles, lungs, and heart. Material properties were assigned based on literature data, and nonlinear contacts were defined among articular and cartilaginous structures. Model validation was carried out using two scenarios: pure-moment loading of the T12–L1 functional spinal unit and a frontal chest impact simulation, with the numerical responses compared against available experimental and cadaveric data.

**Results:**

The T12–L1 moment–rotation curves agreed well with published biomechanical ranges, and the frontal impact simulation produced a peak force (3,270 N) and chest compression (79 mm) closely matching experimental results (3,453 N and 80 mm), with errors of 5.3% and 1.25%, respectively.

**Conclusions:**

The finite element model reproduced static and dynamic responses of the spine–thorax complex within available experimental ranges for the loading conditions examined, providing an initial, non-invasive platform for investigating load transmission, coupled motion, and stress distribution under physiological, pathological, and interventional conditions.

## Introduction

Spinal and thoracic disorders are major contributors to pain, disability, and health-care burden worldwide (Cheng et al., 2025). The mechanical behavior of the spine–thorax complex results from strongly coupled interactions among vertebrae, intervertebral discs, ribs, costal cartilage, the sternum, ligaments, trunk muscles, and intrathoracic organs (Carpenedo et al., 2025). A detailed understanding of whole spine–thorax biomechanics is therefore essential for elucidating the pathogenesis of spinal deformities and degenerative diseases and for guiding surgical reconstruction, implant design, protection against impact injury, and rehabilitation strategies (Xu et al., 2025).

Manual therapy, as a key component of both traditional and contemporary rehabilitation, plays a crucial role in alleviating musculoskeletal pain, restoring joint mobility, and enhancing neuromuscular coordination. Its outcomes depend on mechanical parameters such as the magnitude, rate, direction, and duration of the applied force, as well as on how external loads interact with internal spinal structures. Experimental studies using animal models, cadavers, and human volunteers have improved our understanding of these mechanisms but face important limitations: animals differ markedly from humans in anatomy and tissue mechanics; cadaveric testing cannot reproduce dynamic physiological responses and is constrained by specimen availability; and human studies must respect strict safety thresholds, limiting the exploration of high-load conditions relevant to manipulative procedures (Frantsuzov et al., 2023; Gupta et al., 2020; Pan et al., 2023). These constraints highlight the need for a non-invasive, reproducible, and anatomically accurate method to quantitatively simulate spine–thorax mechanics under controlled conditions.

Computational biomechanics, particularly finite element (FE) modelling, provides such a framework. FE methods discretise the human body into elements with tissue-specific material properties, enabling systematic evaluation of how complex loads influence deformation, stress distribution, and structural stability. Unlike empirical testing, FE analysis permits repeatable, parameter-controlled simulations of mechanical environments that would otherwise be infeasible or unsafe, and has become an essential tool in orthopaedic biomechanics and rehabilitation research (Xu et al., 2020; Xue et al., 2021). Over the past decades, numerous thoracic FE models have been developed for injury biomechanics, automotive safety, and defence applications. Early models (Roberts & Chen, 1970; Sundaram & Feng, 1977) used simplified rib and sternum geometries under static loads, whereas later work (Roth et al., 2013; Shen et al., 2008) incorporated ribs, costal cartilage, and internal organs based on computed tomography (CT) or Visible Human data. More advanced human body models, such as the Ford Human Body Model, the HUBYX model, and the GHBMC series (El-Jawahri et al., 2010; Roth et al., 2013; Zeng et al., 2021), further improved biological realism through multi-tissue representation and dynamic validation, with continued refinements in mesh quality, age-specific modelling, and complex load simulation (Antona-Makoshi et al., 2015; Chaufer et al., 2023; Li et al., 2017). However, most thoracic FE models were developed for impact biomechanics rather than therapeutic or rehabilitation contexts, often simplifying the rib–spine junction and omitting critical spinal components such as intervertebral discs and ligaments. Conversely, many spinal FE models exclude the thoracic cage and surrounding soft tissues, which are crucial for distributing forces across the upper body. This methodological gap limits accurate simulation of coupled thoracic–spinal motion and load transfer pathways during manual therapy and other interventions.

To address these shortcomings, the present study aimed to establish and validate a three-dimensional finite element model of the entire spine–thorax complex of a Chinese adult male, constructed directly from high-resolution CT data. We hypothesized that (i) under quasi-static pure-moment loading, the model would reproduce T12–L1 moment–rotation responses (flexion–extension, lateral bending, and axial rotation) within published experimental ranges; and (ii) under a representative frontal thoracic impact condition, the model would predict peak impact force and peak chest compression comparable to cadaveric benchmarks, with relative errors within an a priori acceptable range (*e.g.*, <10%).

## Materials & Methods

### CT scan of human body

One healthy young adult Chinese male volunteer with typical Chinese male anthropometric characteristics (chest circumference and body mass index) was recruited and provided written informed consent to participate in the study. These anthropometric characteristics fall within the range of a mid-sized adult male, providing reasonable comparability with experimental thoracic impact data, which are typically obtained from mid-sized adult specimens. In addition to a general physical examination, a spine specialist performed a detailed assessment of spinal alignment and posture and reviewed whole-spine imaging to exclude structural deformities, including scoliosis, abnormal thoracic kyphosis or lumbar lordosis, vertebral fractures, tumors, and other pathologies. The volunteer’s sagittal and coronal spinal alignment parameters were within the normal range for an age- and sex-matched healthy adult, and no segmental malalignment was identified.

Subsequently, the entire spine (cervical, thoracic, lumbar, and sacral regions) and thorax were scanned using a 64-slice CT scanner (Aquilion, Canon Medical Systems, Otawara, Tochigi, Japan) with the subject in a neutral, relaxed supine posture to obtain complete spinal and thoracic CT images for model construction. The images were exported in DICOM format, yielding a total of 1030 DICOM images for subsequent modeling. Details of the software used for image processing and model generation are summarized in Table 1. The study protocol was approved by the Institutional Review Board of the Affiliated Traditional Chinese Medicine Hospital, Guangzhou Medical University (approval no. 2025NK38; date: April 11, 2025).

**Table 1:** Main instruments and equipment.

	Corporation	Country
Computed Tomography	Canon Medical Systems	Japan
Mimics Research 21.0	Materialise	Belgium
Geomagic Wrap 2021	3D Systems	United States of America
SolidWorks 2022	Dassault Systèmes	France
Ansys Workbench 2023	Ansys	United States of America

### Geometric reconstruction of human body model

#### Segmentation in mimics research 21.0

CT image data were imported into Mimics Research 21.0 (Materialise, Leuven, Belgium) for three-dimensional reconstruction of the spinal and thoracic structures. Automated thresholding and region-growing segmentation were used to isolate the spine, ribs, lungs, and heart, followed by manual refinement to remove noise and repair voids caused by CT intensity overlap. Individual skeletal elements were then separated and corrected to ensure anatomical accuracy, and the final segmented structures were exported in stereolithography (STL) format for subsequent processing.

#### Surface generation in Geomagic Wrap 2021

The STL files were then imported into Geomagic Wrap 2021 (3D Systems, Rock Hill, SC, USA) for surface optimization. Mesh artifacts were automatically detected and corrected, and surface smoothing was performed to improve topology while preserving anatomical landmarks. A high-quality, closed non-uniform rational B-spline (NURBS) surface model was generated to ensure continuity and geometric precision of the thoracic and spinal components. The finalized geometry was then exported in the standard for the exchange of product data (STP) format for downstream applications.

#### Development of solid models in SolidWorks 2022

Each segmented anatomical structure was imported into SolidWorks 2022 (Dassault Systèmes, Vélizy-Villacoublay, France) to generate the corresponding solid components. For key osseous structures such as the vertebrae, ribs, and sternum, cortical and cancellous bone regions were differentiated. The intervertebral discs—including the nucleus pulposus, annulus fibrosus, and cartilage endplates—were reconstructed, with the nucleus pulposus occupying approximately 44% of the total disc volume. Costal cartilage was modelled in SolidWorks according to its anatomical morphology. Additional anatomical features, including the major thoracic ligaments, lungs, and heart, were also represented. All components were then assembled to form an integrated anatomical model suitable for biomechanical analysis. The primary articulations of the spine and thoracic region include the facet joints, costovertebral joints, sternocostal joints, and costotransverse joints. Facet joints were constructed by defining the articular cartilage between adjacent vertebrae, specifically between the lateral surface of the inferior articular process of the superior vertebra and the medial surface of the superior articular process of the inferior vertebra.

**Figure 1:**
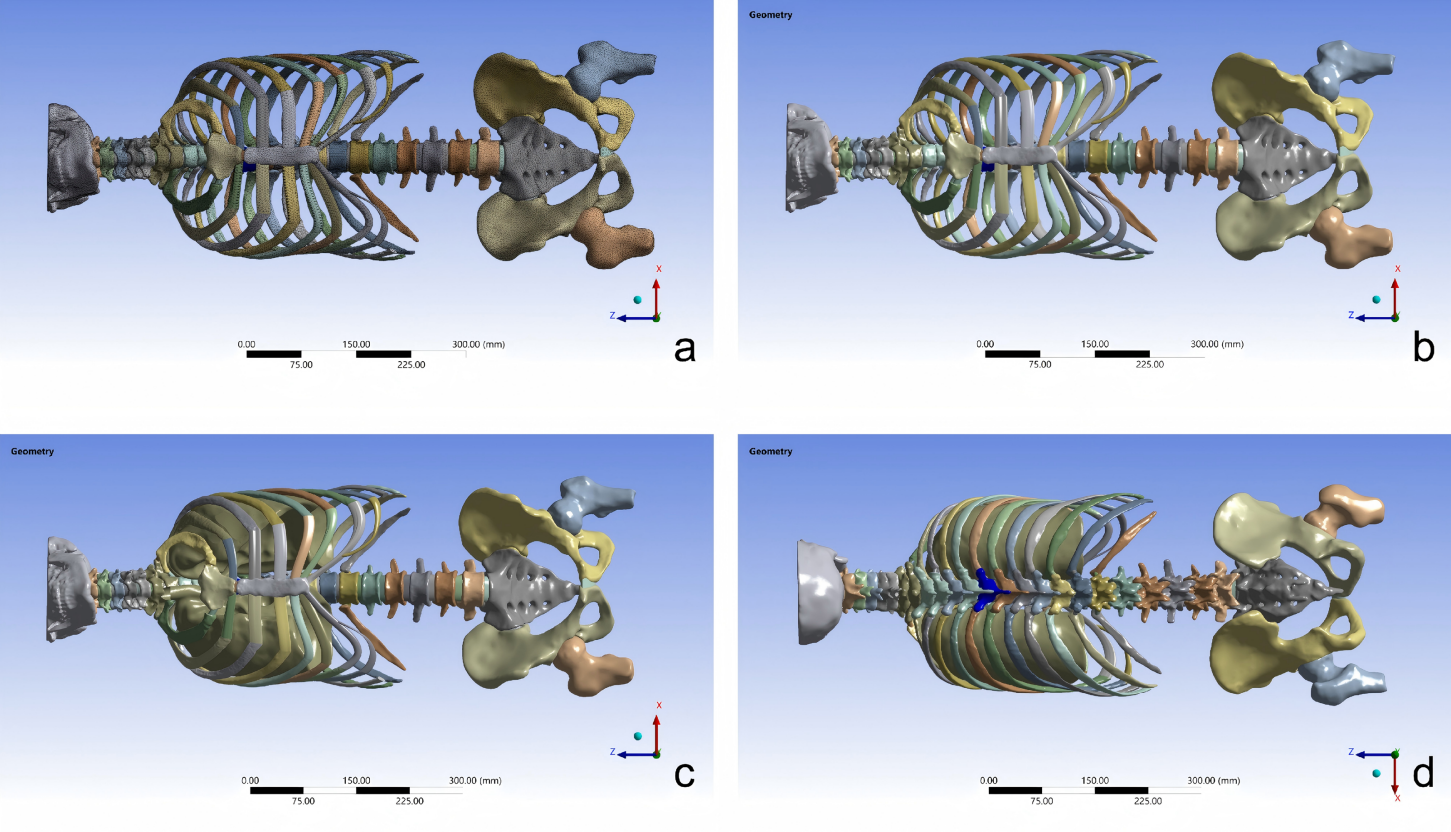
Assembly of the full-spine and thoracic 3D finite element model: (A) full spine and thorax with costal cartilage (mesh); (B) full spine and thorax with costal cartilage; (C) model with lungs assembled; (D) model with major respiratory muscles assembled.

Following the method of Zhang et al. (2016), the main respiratory muscles were constructed in the model. In particular, the diaphragm was reconstructed as a dome-shaped musculotendinous sheet spanning the lower ribs, costal cartilage, sternum, and lumbar attachments, partitioning the thoracic and abdominal cavities and providing a continuous load path between them. The attachment sites and fiber orientations of the diaphragm, external intercostal muscles, and internal intercostal muscles were determined with reference to anatomical textbooks and cadaveric dissections (Fig. 1). After assembly, the “Interference Check” function in SolidWorks was used to identify potential interferences between components. Once all detected interferences had been resolved, the finalized model was saved and exported in Parasolid text transmittal (X_T) format.

#### Development of the finite element model

The assembled model was imported into ANSYS Workbench 2023 for finite element analysis and post-processing. Tissue-specific material properties for each anatomical structure were defined in the Engineering Data module according to values reported in the literature (Zhang et al., 2016; Zhang et al., 2020) and are summarized in Tables 2 and 3. These properties—including elastic modulus, Poisson’s ratio, density, and the corresponding constitutive laws—were then assigned in the Mechanical module to the vertebrae, intervertebral discs, ribs, costal cartilage, sternum, ligaments, muscles, and thoracic organs.

**Table 2:** Material parameters of the main structures of the full spine including the thorax model.

Framework	Isomorphism	Density ρ (kg/m^3^)	Young’s modulus E (MPa)	Poisson’s ratio	Yield stress (MPa)
Vertebral cortical bone	Elastic	2,500	12,000	0.3	
Vertebral cancellous bone	Elastic	1,000	345	0.2	
Annulus fibrosus	Elastic	1,040	300	0.4	
Nucleus pulposus	Elastic	1,040	0.2	0.499	
Sternal cortical bone	Elastic–plastic	2,000	10,000	0.3	90
Sternal cancellous bone	Elastic–plastic	1,000	40	0.45	1.8
Costal cartilage	Elastic–plastic	1,000	49	0.4	4.9
Costal cortical bone	Elastic–plastic	2,000	10,000	0.3	90
Costal cancellous bone	Elastic–plastic	1,000	40	0.45	1.8
Anterior longitudinal ligament	Elastic–plastic	1,000	20	0.22	
Posterior longitudinal ligament	Elastic–plastic	1,000	9.12	0.40	
Intertransverse ligament	Elastic–plastic	1,000	10	0.3	
Ligamentum flavum	Elastic–plastic	1,000	10	0.40	
Interspinous ligament	Elastic–plastic	1,000	10	0.4	
Supraspinous ligament	Elastic–plastic	1,000	8	0.3	
Articular cartilage	Elastic–plastic	1,000	49	0.4	4.9
Joint capsule	Elastic–plastic	1,000	10	0.4	

**Table 3:** Viscoelastic and elastic material properties of the thoracic tissue.

Framework	Material properties	Density ρ (kg/m^3^)	Volumetric Modulus of Elasticity (MPa)	Short-time shear modulus (MPa)	Long-time shear modulus (MPa)	Decay constant
Lungs	Viscoelasticity	600	0.22	0.02	0.75	0.25
Heart	Viscoelasticity	1,000	2.6	0.44	0.15	0.25
Muscles	Resilient	1,200	0.5			

The model was discretized predominantly using three-dimensional solid elements. Mesh density was increased in regions of high geometric complexity (*e.g.*, vertebral arches, costovertebral joints, and rib angles) and reduced in more uniform regions to balance accuracy and computational cost. To limit the overall model size while retaining the global mass and bulk compliance of the thoracic cavity, the lungs were represented using shell elements with an assigned effective thickness and density, so that their contribution to overall inertia and stiffness was preserved without resolving detailed intraparenchymal stress fields. Mesh quality was evaluated using standard criteria (element aspect ratio and skewness), and elements that did not meet the predefined quality thresholds were locally refined or remeshed to avoid compromising the numerical stability and accuracy of the simulations.

Material properties were assumed to be continuous, homogeneous, and isotropic unless otherwise specified. The lungs and heart were assigned homogeneous, isotropic viscoelastic properties based on reported bulk and shear relaxation moduli for thoracic soft tissues, so as to capture their time-dependent response under dynamic loading. The diaphragm and intercostal muscles, together with the other thoracic muscles, were meshed using three-dimensional solid elements and modeled as isotropic linear elastic soft tissues to represent their passive bulk and compressive/shear stiffness; no Hill-type active contraction or force–length–velocity behavior was included in the present simulations. The corresponding viscoelastic parameters for the lungs and heart and the linear elastic parameters for the thoracic muscles were taken from previously published thoracic finite element studies (Zhang et al., 2016; Zhang et al., 2020) and are listed in Tables 2 and 3. Three-dimensional nonlinear contact interactions were defined at the costosternal joints, costovertebral joints, facet joints, and their associated cartilaginous structures under frictionless contact conditions. Interactions between vertebrae and intervertebral discs, as well as between vertebrae and ligaments, were modeled as bonded contacts (Wang, 2020).

**Figure 2:**
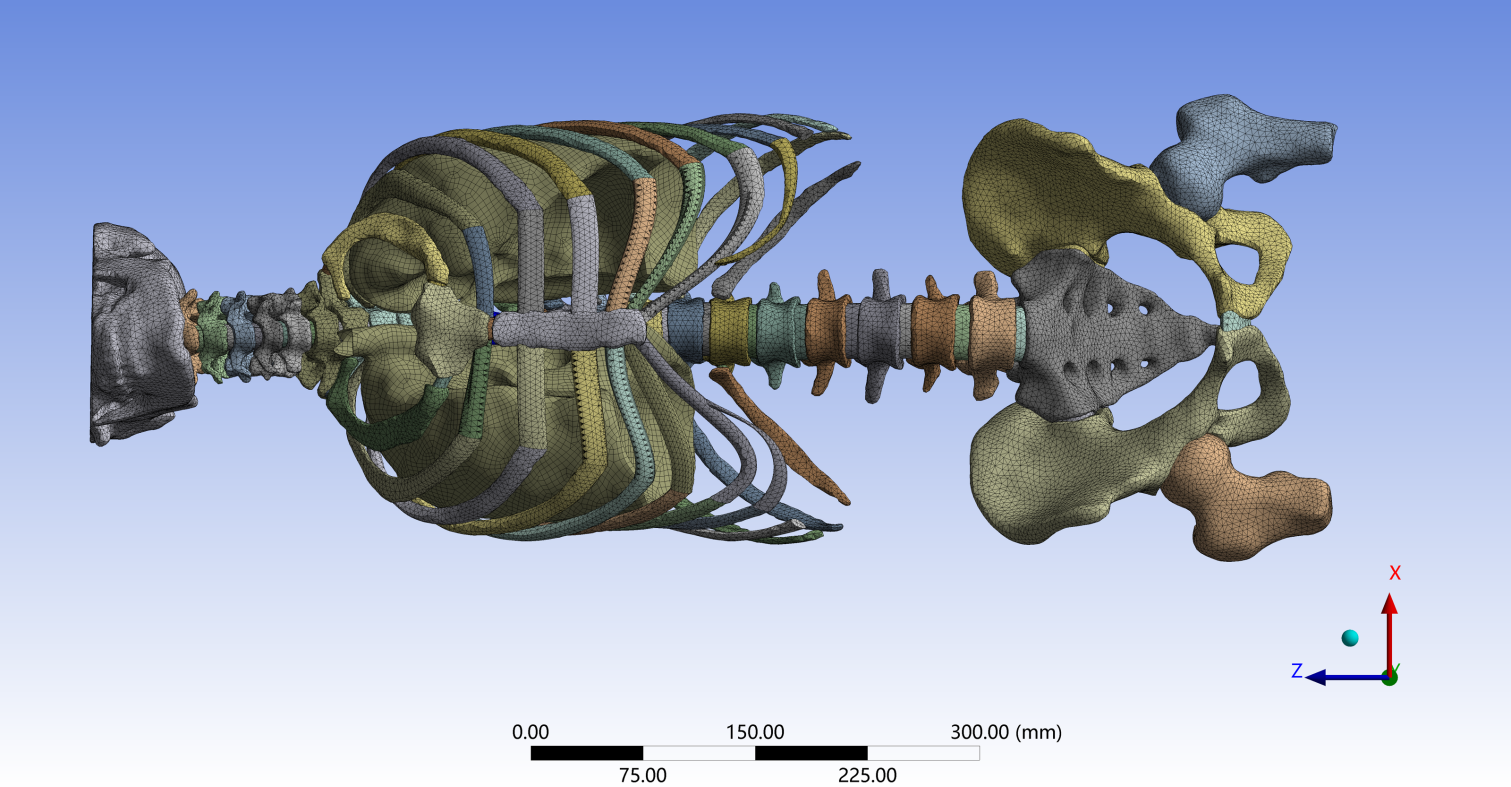
Simulation setup for frontal chest collision validation of the finite element model.

A three-dimensional finite element model of the entire spine, including the thoracic region, was thereby established (Fig. 2). The final model consisted of 374,852 nodes and 470,524 elements. It incorporated multiple anatomical structures, including the inferior portion of the skull, vertebrae (cortical and cancellous bone), sacrum, ilium, proximal femur, annulus fibrosus, nucleus pulposus, sternum, costal cartilage, ribs, major spinal ligaments, articular cartilage, joint capsules, lungs, heart, and respiratory muscles. This comprehensive anatomical representation provides a detailed structural basis for simulating the biomechanical behavior of the spine–thorax complex.

### Validation of the finite element model

The model was evaluated under two representative loading conditions to provide initial validation of its mechanical behavior. First, a pure-moment loading protocol was applied to the T12–L1 functional spinal unit to obtain moment–rotation responses in six primary motion directions: spinal flexion, extension, left and right lateral bending, and left and right axial rotation. These responses were compared with data from previously published *in vitro* studies. Second, a frontal chest impact of the whole spine–thorax model was simulated using a rigid wooden cylindrical impactor (diameter 15.24 cm, mass 23.4 kg) striking the thorax at velocities of 4, 6, and 8 m/s. The resulting peak global impact force and peak chest compression were then compared with cadaveric and other published experimental data.

#### Validation of the spine

In biomechanical studies of the spine, two adjacent vertebrae and their intervertebral connective soft tissues are commonly referred to as the functional spinal unit (FSU) or motion segment (Chow & Gregory, 2023). The anterior portion of the FSU consists primarily of the anterior longitudinal ligament, vertebral body, intervertebral disc (nucleus pulposus and annulus fibrosus), and posterior longitudinal ligament. The posterior portion is mainly composed of the ligamentum flavum, vertebral arch (pedicle and lamina), facet joints, transverse processes (with costovertebral joints in the thoracic spine), spinous processes, and associated ligaments (interspinous ligament, supraspinous ligament, and intertransverse ligament).

The FSU is the smallest functional unit that exhibits biomechanical characteristics similar to those of the entire spine. Studies have shown that when loads are applied to the FSU, three-dimensional motion with six degrees of freedom can be achieved, comprising three translational displacements and three rotational angles. The T12–L1 motion segment was selected as the representative analysis region because it lies at the transitional junction between the thoracic and lumbar spines, where load transfer and mobility characteristics are biomechanically critical. This segment has been widely used in previous validation studies to evaluate the mechanical fidelity of finite element spine models (Li et al., 2025; Qiu et al., 2006). Moreover, due to its intermediate anatomical position and the availability of experimental reference data, T12–L1 provides a suitable benchmark for assessing model accuracy under flexion–extension and axial rotation.

**Figure 3:**
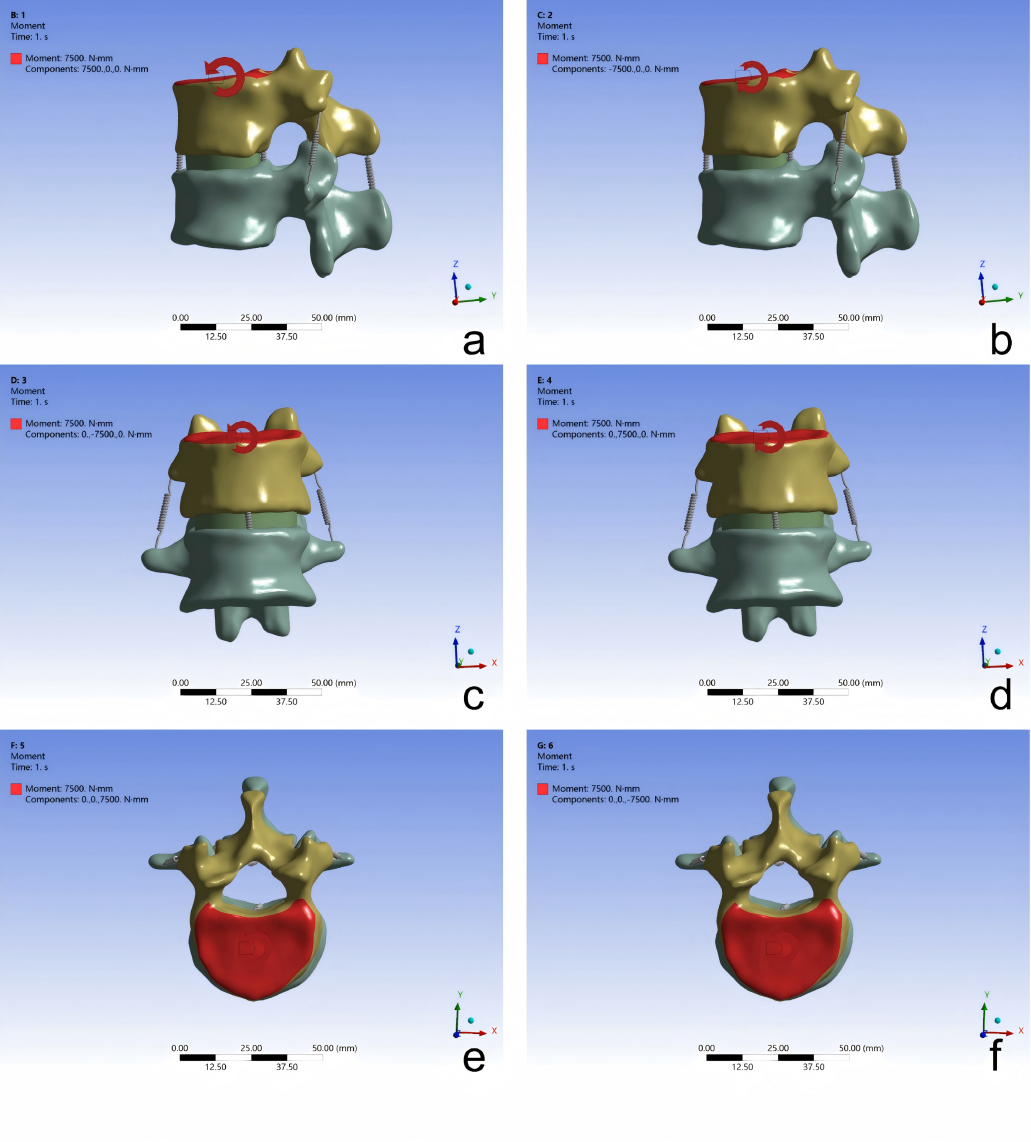
Static validation of the T12-L1 functional spinal unit 3D finite element model: (A) flexion; (B) extension; (C) left lateral bending; (D) right lateral bending; (E) left rotation; (F) right rotation.

A right-handed, thumb-up Cartesian coordinate system was adopted, with the geometric center of the superior vertebra selected as the origin. The inferior surface of the L1 vertebral body was fully constrained in all six degrees of freedom. The superior endplate of the T12 vertebra was kinematically coupled to a reference point located at its geometric center. A pure moment of 7.5 N⋅m was applied incrementally at this reference point about the global X, Y, or Z axis to simulate six motion scenarios: spinal flexion, extension, left lateral bending, right lateral bending, left axial rotation, and right axial rotation (Zhang et al., 2021) (Fig. 3). The resulting ranges of motion were compared with those reported in published experimental studies for validation.

#### Validation of chest frontal collision

This validation procedure was based on cadaveric frontal-impact experiments reported in previous studies (Kroell, Schneider & Nahum, 1971; Kroell, Schneider & Nahum, 1974), which have been widely used as benchmarks for thoracic finite element model validation. In this study, the whole spine–thorax finite element model was subjected to loading conditions (impactor mass, initial velocity, impact direction, and support configuration) consistent with these experiments. Nodes on the posterior aspects of the thoracic vertebral bodies and adjacent ribs were kinematically coupled to a rigid back plate representing the experimental support surface; this plate was fixed in all translational and rotational degrees of freedom to prevent rigid-body motion while allowing local deformation of the chest wall.

**Figure 4:**
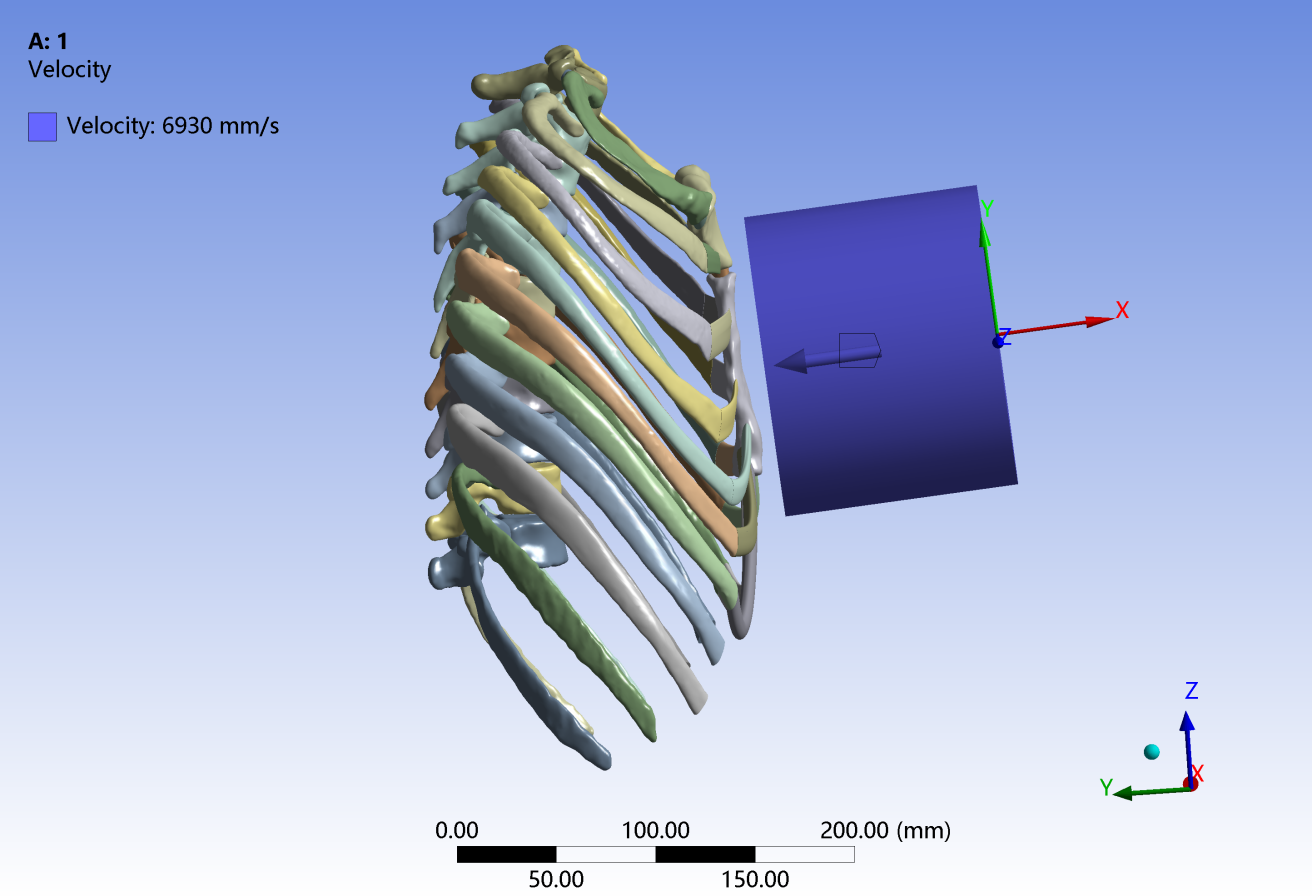
Three-dimensional finite element model of the entire spine including the thorax for an adult Chinese male developed in this study.

A rigid wooden cylindrical impactor (diameter 15.24 cm, mass 23.4 kg) was positioned anterior to the mid-sternum and given an initial velocity of 6.93 m/s along the anteroposterior axis. The elastic modulus and density of the cylinder were defined according to the material properties of wood to ensure consistency with the experimental conditions (Schoell et al., 2015). Automatic surface-to-surface contact with a friction coefficient of 0.3 was defined between the impactor and the anterior chest wall (Fig. 4). Because the CT-based volunteer represents a mid-sized adult male with anthropometry broadly comparable to the experimental specimens, no additional anthropometric scaling of the impact responses was applied, and all comparisons were performed in absolute terms. Model validation was carried out by comparing the simulated peak global impact force and peak chest compression with the corresponding cadaveric values, as these two metrics are widely used indicators of thoracic injury severity in frontal impact studies. Due to the output configuration adopted in the original simulations, only the peak responses were stored, and the complete force–time and chest-displacement–time histories could not be retrieved for quantitative comparison.

### Statistical analysis

This study was based on deterministic finite element simulations of a single subject-specific whole spine–thorax model. Consequently, no formal sample size calculation or inferential hypothesis testing was performed.

## Results

### Subject characteristics

The finite element model was reconstructed from CT data of a healthy adult Chinese male (25 years, 175 cm, 70 kg). Clinical and imaging screening indicated no evidence of spinal or thoracic trauma, deformity, scoliosis, tumor, or other structural pathology.

### Validation results of the model

#### Static validation of the spine

**Figure 5:**
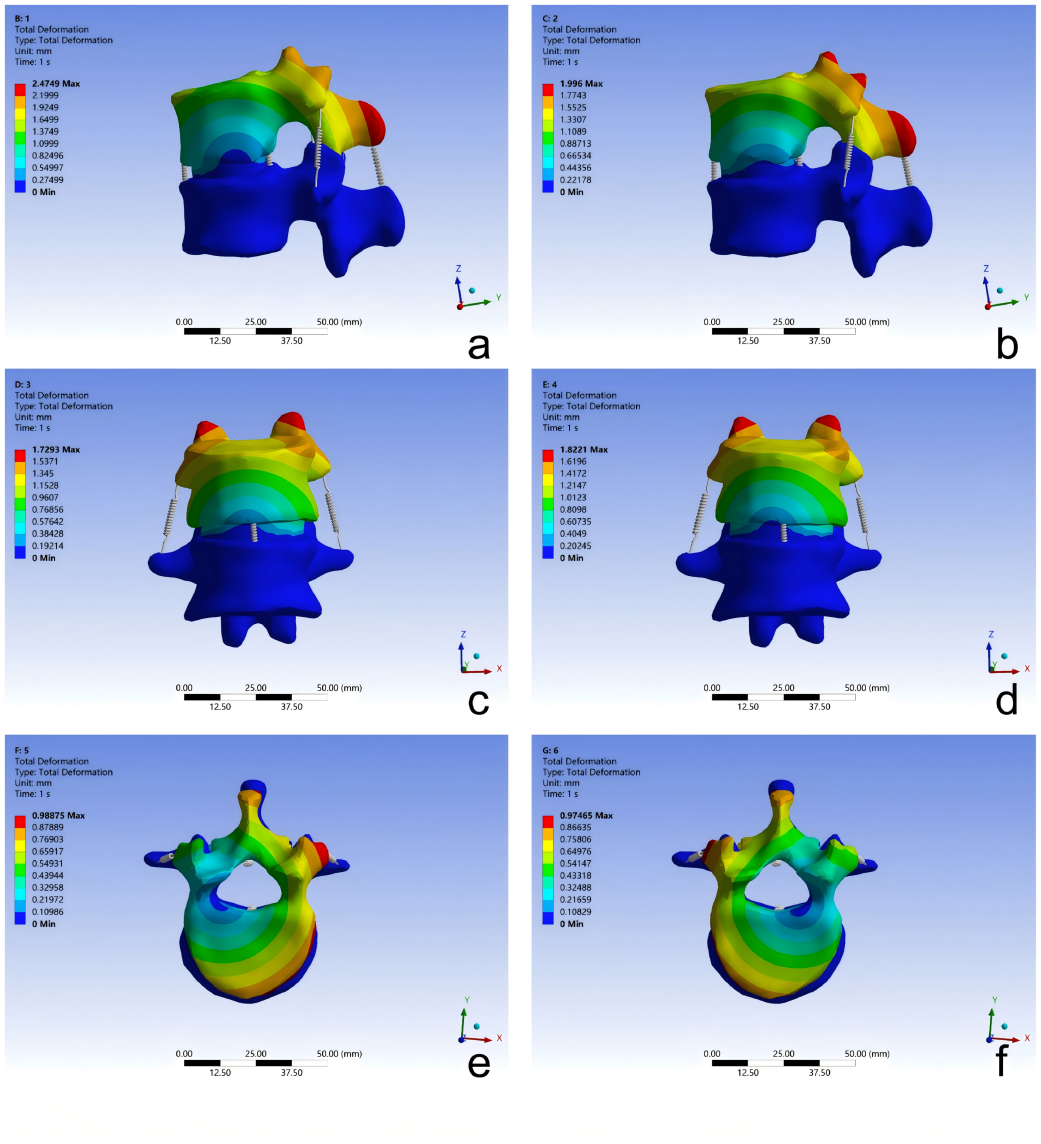
Total deformation contour plot of the T12–L1 functional spinal unit 3D finite element model under static validation: (A) flexion; (B) extension; (C) left lateral bending; (D) right lateral bending; (E) left axial rotation; (F) right axial rotation.

**Figure 6:**
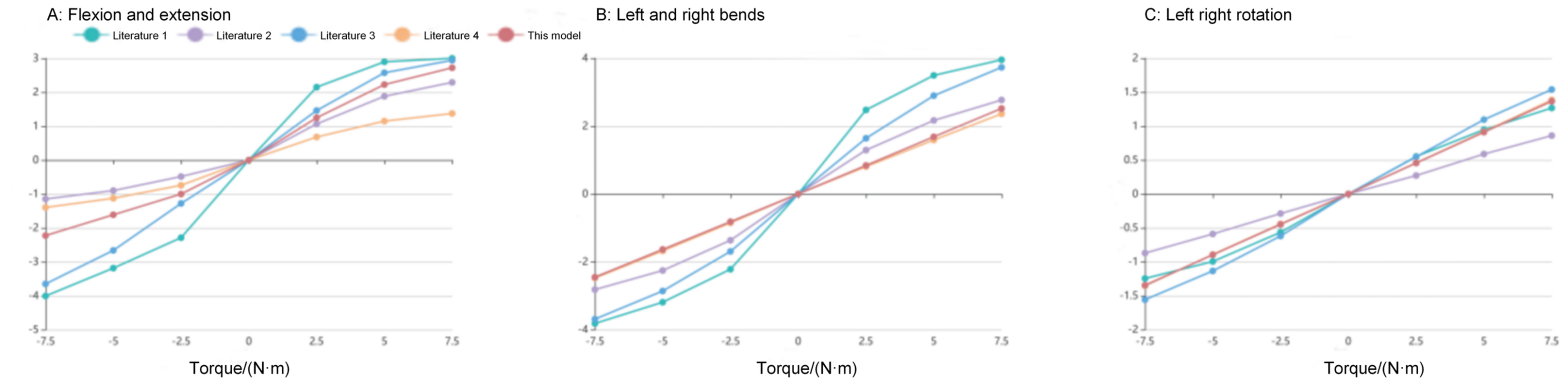
Load–rotation curves of the T12–L1 finite element model under different moments compared with experimental data from the literature (Zhang et al., 2021; Oxland, Lin & Panjabi, 1992; Markolf, 1972; Qiu, 2005): (A) flexion–extension; (B) left and right lateral bending; (C) axial rotation.

The angular displacements of the T12–L1 finite element model under six pure-moment loading conditions were compared with previously reported *in vitro* data (Markolf, 1972; Oxland, Lin & Panjabi, 1992; Qiu, 2005; Zhang et al., 2021). The model exhibited a monotonic increase in rotation with increasing applied moment, consistent with the load–rotation behavior described in these experimental studies. For flexion, extension, lateral bending, and axial rotation, the predicted peak angular displacements remained within the corresponding experimental ranges (with positive and negative values denoting opposite directions of motion). These findings indicate that the simulated moment–rotation curves of the T12–L1 segment are in good agreement with published data (Figs. 5 and 6).

#### Frontal chest collision validation

**Figure 7:**
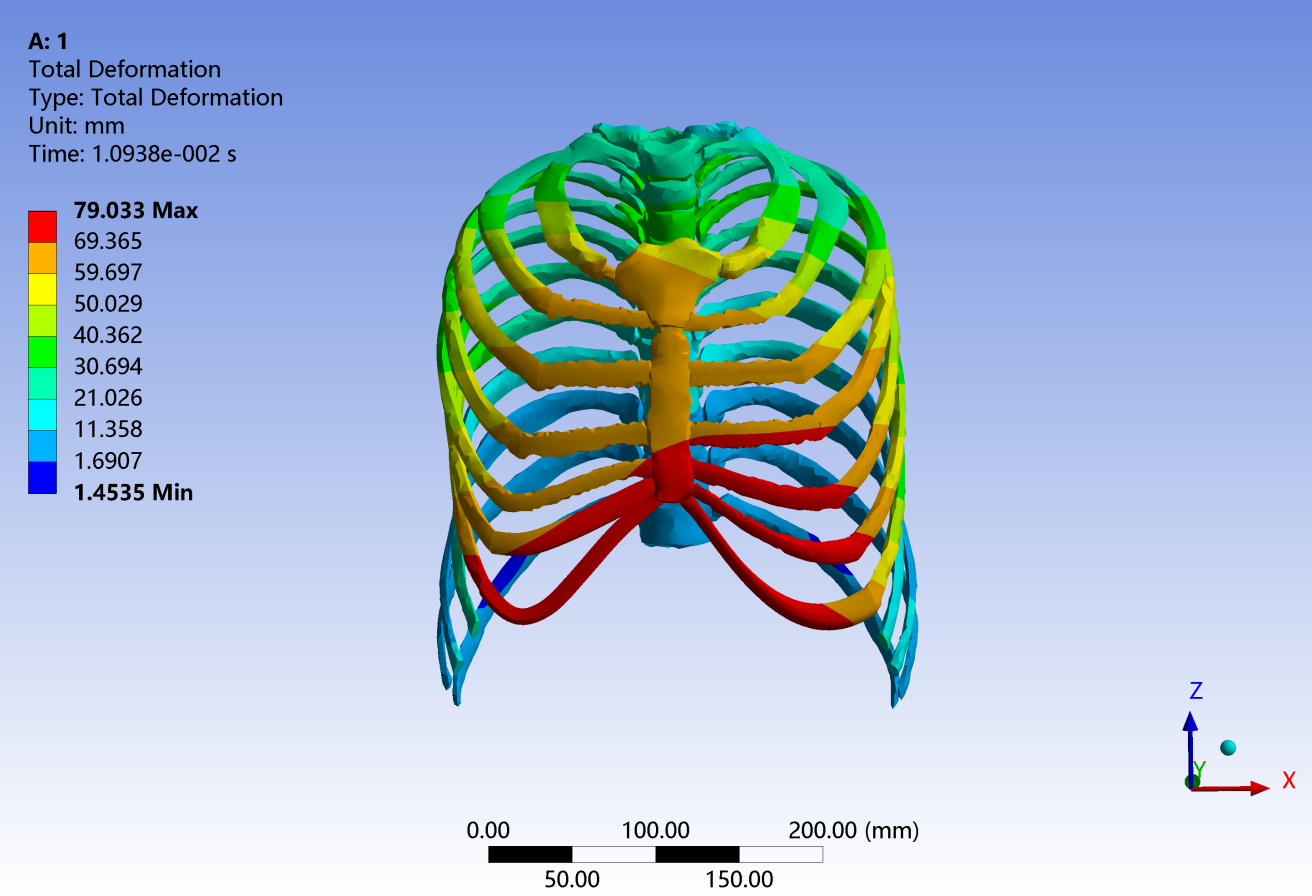
Total deformation contour of the thorax during frontal chest impact validation.

During the frontal chest impact simulation, the cylindrical impactor decelerated after contacting the chest skin and muscles, leading to a progressive increase in contact force. No rib or sternal fracture was predicted under the current material and failure assumptions. In the cadaveric frontal impact tests, the peak impact force was 3,453 N, whereas the finite element model predicted a peak force of 3,270 N, corresponding to a difference of 5.3%. Similarly, the peak chest compression measured in the cadaveric tests was 80 mm, while the simulation yielded 79 mm, with an error of 1.25% (Kroell, Schneider & Nahum, 1971; Kroell, Schneider & Nahum, 1974; Wang, 2020) (Fig. 7 and Table 4). Figure 7 illustrates the distribution of total thoracic deformation at the time of peak chest compression during the frontal impact. In the present work, quantitative validation of the model is therefore based on these peak responses (peak force and peak chest compression), which show good agreement with the cadaveric data. Both errors were below the pre-defined acceptable threshold (<10%).

## Discussion

This study describes the development of a high-fidelity, whole spine–thorax finite element model reconstructed from high-resolution CT of a healthy adult male and its initial validation under two representative loading conditions. The T12–L1 functional spinal unit produced moment–rotation relationships for flexion–extension, lateral bending, and axial rotation that fell within ranges reported in previous *in vitro* experiments (Li, Shen & Li, 2017; Yu et al., 2025). The simulated frontal impact yielded a peak force of 3,270 N and a peak chest compression of 79 mm, closely matching cadaveric data (3,453 N and 80 mm, respectively), with differences of approximately 5.3% and 1.25%. These agreements suggest that an anatomically explicit, multi-tissue representation of the spine–thorax complex can capture key kinematic and impact responses for the specific loading conditions examined, providing preliminary support for its use in whole spine–thorax biomechanical analysis.

**Table 4:** Results of frontal chest collision test verification.

**Validation criteria**	**This model**	**Reference model**	**Cadaveric experiment**	**Error between this model and the cadaveric experiment**
Peak collision force	3,270 N	3,120 N	3,453 N	5.3%
Chest compression	79 mm	75 mm	80 mm	1.25%

The principal contribution of this work is the integration of detailed thoracic anatomy with a segmental spinal representation within a single computational framework. Whereas prior models have typically focused on either thoracic impact and injury mechanics or isolated spinal kinematics (Meyer et al., 2021; Singh et al., 2024), the present model explicitly represents costovertebral and costotransverse articulations, intervertebral discs with constituent regions, major ligaments, articular cartilage, and primary respiratory muscles, assembled into a mesh of sufficient resolution to permit coupled analyses. This integrated geometry makes it possible to examine load pathways and coupled motion patterns that are difficult to quantify with separate or overly simplified models and are relevant to a variety of interventional scenarios, including manual therapeutic procedures (Naoum et al., 2021). The dual-mode validation presented here—comparing both segmental pure-moment behavior and a dynamic impact response against experimental benchmarks—provides complementary evidence that the model can represent both low-energy manipulative mechanics and higher-energy loading regimes that interrogate chest structural response (Wiczenbach et al., 2023).

From an application standpoint, the integrated model offers several practical uses. It enables controlled, repeatable exploration of how the magnitude, direction, velocity, and duration of external inputs influence internal stresses and motions, and can be used to identify loading regimes that concentrate stress in vulnerable structures. Comparative simulations that intentionally simplify or remove specific anatomical components can reveal which features materially affect outcomes and therefore where modeling fidelity is most critical for particular research questions (Hatano et al., 2014; Hatano et al., 2013; Nagasao et al., 2016). To move computational findings toward clinically relevant guidance, the model could be combined with instrumented clinical measurements—such as force-sensing gloves, synchronized motion capture, and surface electromyography—so that applied inputs can be mapped to predicted internal loads and physiological responses (Lin et al., 2023; Wan et al., 2025). Ultimately, a cohort of subject-specific or parametric models reflecting different ages, sexes, and common pathologies, together with probabilistic sensitivity analyses, will be helpful to quantify how patient variability modulates biomechanical risk and to progress from case-based simulations toward more generalizable insights.

Several limitations should also be acknowledged. First, the validation performed in this study is necessarily limited in scope: it focuses on a single thoracolumbar motion segment (T12–L1) under pure-moment loading and on a single frontal chest impact configuration, and therefore does not constitute exhaustive validation across all spinal levels, postures, or loading scenarios. Second, the thoracic impact validation was carried out using the absolute peak global impact force and peak chest compression, without formal normalization for inter-subject anthropometric differences between the finite element model and the cadaveric specimens. Although the volunteer’s height and body mass fall within the range of a mid-sized adult male, more rigorous anthropometric scaling and parametric modeling across different body sizes would further strengthen the generalizability of the impact validation. Third, the model was derived from CT data of a single healthy young male with clinically normal sagittal and coronal spinal alignment and without common spinal pathologies; as such, it does not capture the wide anatomical and material variability associated with sex, age, body habitus, deformity, or degeneration (*e.g.*, disc degeneration, osteoporosis, or scoliosis) (Mangado et al., 2016). In addition, several modeling simplifications were introduced for tractability: many tissues were treated as homogeneous and isotropic or represented by idealized nonlinear constitutive laws; muscle tissue, including the diaphragm and intercostal muscles, was modeled passively without physiological activation, intra-abdominal pressure generation, or reflex dynamics; contact at several interfaces was assumed frictionless or otherwise simplified; and some structures, most notably the lungs, were discretized with shell elements rather than three-dimensional solid elements. While this simplification was sufficient for reproducing the global impact force and chest compression within experimental ranges in the tested scenarios, it may under-represent intrathoracic pressure distribution and through-thickness stress gradients and thus is not suitable for detailed studies of lung deformation or parenchymal injury. Future work should therefore incorporate anthropometric scaling, subject-specific or population-based model cohorts, active muscle representations, more refined constitutive and contact formulations, and expanded validation across multiple spinal regions, loading conditions, and malaligned postures (*e.g.*, hyperkyphosis, hypolordosis, or scoliosis).

## Conclusions

This study developed a high-fidelity three-dimensional finite element model of the whole spine–thorax complex based on high-resolution CT images of a healthy adult male, integrating vertebrae, intervertebral discs, ribs, costal cartilage, sternum, major ligaments, key trunk muscles, and representative thoracic organs. The model reproduced segmental moment–rotation behavior and global frontal impact responses within available experimental ranges for the loading conditions examined, providing preliminary support for its use in whole spine–thorax biomechanical analysis. It offers a quantitative platform for studying load transmission, coupled motion, and stress distribution under a range of physiological, pathological, and interventional conditions, supporting applications in areas such as surgical planning, implant evaluation, injury biomechanics, and rehabilitation research.

## Additional Information and Declarations

### Data Availability

The following information was supplied regarding data availability: The raw data is available at Figshare: - Yu (2026). Finite Element Model. figshare. Dataset. https://doi.org/10.6084/m9.figshare.31190800.v1. - Yu (2026). Development and biomechanical validation of a whole spine–thorax finite element model for quantitative biomechanical analysis. figshare. Dataset. https://doi.org/10.6084/m9.figshare.31190218.v1.

## References

[ref-1] Antona-Makoshi J, Yamamoto Y, Kato R, Sato F, Ejima S, Dokko Y, Yasuki T (2015). Age-dependent factors affecting thoracic response: a finite element study focused on Japanese elderly occupants. Traffic Injury Prevention.

[ref-2] Carpenedo L, Ignasiak D, Remus R, La Barbera L (2025). Advances in musculoskeletal modeling of the thoraco-lumbar spine: a comprehensive systematic review. Annals of Biomedical Engineering.

[ref-3] Chaufer M, Delille R, Bourel B, Marechal C, Lauro F, Mauzac O, Roth S (2023). A new biomechanical FE model for blunt thoracic impact. Frontiers in Bioengineering and Biotechnology.

[ref-4] Cheng M, Xue Y, Cui M, Zeng X, Yang C, Ding F, Xie L (2025). Global, regional, and national burden of low back pain: findings from the global burden of disease study 2021 and projections to 2050. Spine.

[ref-5] Chow N, Gregory DE (2023). The effect of intervertebral disc damage on the mechanical strength of the annulus fibrosus in the adjacent segment. Spine Journal.

[ref-6] El-Jawahri RE, Laituri TR, Ruan JS, Rouhana SW, Barbat SD (2010). Development and validation of age-dependent FE human models of a mid-sized male thorax. Stapp Car Crash Journal.

[ref-7] Frantsuzov R, Mondal S, Walsh CM, Reynolds JP, Dooley D, MacManus DB (2023). A finite element model of contusion spinal cord injury in rodents. Journal of the Mechanical Behavior of Biomedical Materials.

[ref-8] Gupta D, Zubair M, Lalwani S, Gamanagatti S, Roy TS, Mukherjee S, Kale SS (2020). Development and validation of finite element analysis model (FEM) of craniovertebral junction: experimental biomechanical cadaveric study. Spine.

[ref-9] Hatano A, Nagasao T, Cho Y, Shimizu Y, Takano N, Kaneko T, Kishi K (2014). Relationship between locations of rib defects and loss of respiratory function—a biomechanical study. Thoracic and Cardiovascular Surgeon.

[ref-10] Hatano A, Nagasao T, Shimizu Y, Jin H, Kaneko T, Cho Y, Jiang H, Kishi K (2013). A biomechanical study regarding the effect of tissue harvesting from the thorax on its movement during inspiration. Computer Aided Surgery.

[ref-11] Kroell CK, Schneider DC, Nahum AM (1971). Impact tolerance and response of the human thorax. Proceedings of the Fifteenth Stapp Car Crash Conference.

[ref-12] Kroell CK, Schneider DC, Nahum AM (1974). Impact tolerance and response of the human thorax II. Proceedings of the Eighteenth Stapp Car Crash Conference.

[ref-13] Li XF, Kuang JM, Nie SB, Xu J, Zhu J, Liu YH (2017). A numerical model for blast injury of human thorax based on digitized visible human. Technology and Health Care.

[ref-14] Li P, Mu J, Wang Z, Zhang X, Zhang Y, Liu D, Li A (2025). Construction and validation of a U-type finite element model of an osteoporotic vertebral compression fracture. Frontiers in Bioengineering and Biotechnology.

[ref-15] Li L, Shen T, Li YK (2017). A finite element analysis of stress distribution and disk displacement in response to lumbar rotation manipulation in the sitting and side-lying positions. Journal of Manipulative and Physiological Therapeutics.

[ref-16] Lin D, He Z, Weng R, Zhu Y, Lin Z, Deng Y, Yang Y, Tan J, Wang M, Li Y, Huang G, Yu G, Cai D, Huang X, Huang W (2023). Comparison of biomechanical parameters of two Chinese cervical spine rotation manipulations based on motion capture and finite element analysis. Frontiers in Bioengineering and Biotechnology.

[ref-17] Mangado N, Piella G, Noailly J, Pons-Prats J, Ballester M (2016). Analysis of uncertainty and variability in finite element computational models for biomedical engineering: characterization and propagation. Frontiers in Bioengineering and Biotechnology.

[ref-18] Markolf KL (1972). Deformation of the thoracolumbar intervertebral joints in response to external loads: a biomechanical study using autopsy material. Journal of Bone and Joint Surgery. American Volume.

[ref-19] Meyer F, Humm J, Yoganandan N, Leszczynski A, Bourdet N, Deck C, Willinger R (2021). Development of a detailed human neck finite element model and injury risk curves under lateral impact. Journal of the Mechanical Behavior of Biomedical Materials.

[ref-20] Nagasao T, Kasai S, Shimizu Y, Sakamoto Y, Hatano A, Morotomi T, Ogata H, Kishi K (2016). A biomechanical study of relationship between sternum defect patterns and thoracic respiration. Computer Assisted Surgery.

[ref-21] Naoum S, Vasiliadis AV, Koutserimpas C, Mylonakis N, Kotsapas M, Katakalos K (2021). Finite element method for the evaluation of the human spine: a literature overview. Journal of Functional Biomaterials.

[ref-22] Oxland TR, Lin RM, Panjabi MM (1992). Three-dimensional mechanical properties of the thoracolumbar junction. Journal of Orthopaedic Research.

[ref-23] Pan CC, Lee CH, Chen KH, Yen YC, Su KC (2023). Comparative biomechanical analysis of unilateral, bilateral, and lateral pedicle screw implantation in oblique lumbar interbody fusion: a finite element study. Bioengineering.

[ref-24] Qiu T (2005). Finite element analyses of thoracolumbar junction: investigations of instantaneous axes of rotation (IARs) and burst fracture mechanism. PhD thesis.

[ref-25] Qiu TX, Tan KW, Lee VS, Teo EC (2006). Investigation of thoracolumbar T12-L1 burst fracture mechanism using finite element method. Medical Engineering & Physics.

[ref-26] Roberts SB, Chen PH (1970). Elastostatic analysis of the human thoracic skeleton. Journal of Biomechanics.

[ref-27] Roth S, Torres F, Feuerstein P, Thoral-Pierre K (2013). Anthropometric dependence of the response of a thorax FE model under high speed loading: validation and real world accident replication. Computer Methods and Programs in Biomedicine.

[ref-28] Schoell SL, Weaver AA, Vavalle NA, Stitzel JD (2015). Age- and sex-specific thorax finite element model development and simulation. Traffic Injury Prevention.

[ref-29] Shen W, Niu Y, Mattrey RF, Fournier A, Corbeil J, Kono Y, Stuhmiller JH (2008). Development and validation of subject-specific finite element models for blunt trauma study. Journal of Biomechanical Engineering.

[ref-30] Singh NK, Singh NK, Verma R, Diwan AD (2024). Validation and estimation of obesity-induced intervertebral disc degeneration through subject-specific finite element modelling of functional spinal units. Bioengineering.

[ref-31] Sundaram SH, Feng CC (1977). Finite element analysis in the human thorax. Journal of Biomechanics.

[ref-32] Wan C, Shen X, Wu X, Yu C, Shao Y, Zhang R, Shang J, Li J, Zhang Y, Li Y (2025). Assessing the biomechanics of scheuermann’s kyphosis affected thoracolumbar spine in forward flexion at the tissue-level using a finite element model. Scientific Reports.

[ref-33] Wang J (2020). Development of human thorax finite element model and injury investigation under UAV collision condition. Master thesis.

[ref-34] Wiczenbach T, Pachocki L, Daszkiewicz K, Łuczkiewicz P, Witkowski W (2023). Development and validation of lumbar spine finite element model. PeerJ.

[ref-35] Xu B, Li G, Sun X, Yang S, Sun R, Gong Y, Song M (2025). Main elements of current spine biomechanics research: model, installation and test data. Frontiers in Bioengineering and Biotechnology.

[ref-36] Xu Z, Li Y, Zhang S, Liao L, Wu K, Feng Z, Li D (2020). A finite element analysis of sacroiliac joint displacements and ligament strains in response to three manipulations. BMC Musculoskeletal Disorders.

[ref-37] Xue F, Chen Z, Yang H, Chen T, Li Y (2021). Effects of cervical rotatory manipulation on the cervical spinal cord: a finite element study. Journal of Orthopaedic Surgery and Research.

[ref-38] Yu Z, Deng Z, Chen H, Zhang L, Zhao Y, Zhan H, Lin M, Vrionis F, Wang H (2025). Biomechanical effect of chinese manual therapy for cervical spondylotic radiculopathy after percutaneous endoscopic cervical foraminotomy and diskectomy: a finite element study. Clinical Spine Surgery.

[ref-39] Zeng W, Mukherjee S, Caudillo A, Forman J, Panzer MB (2021). Evaluation and validation of thorax model responses: a hierarchical approach to achieve high biofidelity for thoracic musculoskeletal system. Frontiers in Bioengineering and Biotechnology.

[ref-40] Zhang G, Chen X, Ohgi J, Miura T, Nakamoto A, Matsumura C, Sugiura S, Hisada T (2016). Biomechanical simulation of thorax deformation using finite element approach. BioMedical Engineering Online.

[ref-41] Zhang Q, Chon T, Zhang Y, Baker JS, Gu Y (2021). Finite element analysis of the lumbar spine in adolescent idiopathic scoliosis subjected to different loads. Computers in Biology and Medicine.

[ref-42] Zhang W, Zhao J, Jiang X, Li L, Yu C, Zhao Y, Si H (2020). Thoracic vertebra fixation with a novel screw-plate system based on computed tomography imaging and finite element method. Computer Methods and Programs in Biomedicine.

